# Early Urinary Biomarkers in Pediatric Autosomal Dominant Polycystic Kidney Disease (ADPKD): No Evidence in the Interest of Urinary Neutrophil Gelatinase-Associated Lipocalin (uNGAL)

**DOI:** 10.3389/fped.2019.00088

**Published:** 2019-03-22

**Authors:** Marianthi Tsingos, Laura Merlini, Marco Solcà, Alexandra Goischke, Alexandra Wilhelm-Bals, Paloma Parvex

**Affiliations:** ^1^Pediatric Nephrology Unit, Department of Pediatrics, Children's Hospital, Geneva University Hospital, Geneva, Switzerland; ^2^Pediatric Radiology Unit, Department of Radiology, Children's Hospital, Geneva University Hospital, Geneva, Switzerland; ^3^Laboratory of Cognitive Neuroscience, Brain Mind Institute and Center for Neuroprosthetics, School of Life Sciences, École Polytechnique Fédérale de Lausanne, Geneva, Switzerland

**Keywords:** ADPKD, uNGAL, children, renal impairment, TKV

## Abstract

**Background:** Autosomal Dominant Polycystic Kidney Disease (ADPKD) is increasingly diagnosed during childhood by the presence of renal cysts in patients with a positive familial history. No curative treatment is available and early detection and diagnosis confronts pediatricians with the lack of early markers to decide whether to introduce renal-protective agents and prevent the progression of renal failure. Neutrophil Gelatinase-Associated Lipocalin (NGAL) is a tubular protein that has been recently proposed as an early biomarker of renal impairment in the ADPKD adult population.

**Methods:** Urinary NGAL (uNGAL) levels were measured in 15 ADPKD children and compared with 15 age and gender matched controls using parametric, non-parametric, and Bayesian statistics. We also tested the association of uNGAL levels with markers of disease progression, such as proteinuria, albuminuria, blood pressure, and Total Kidney Volume (TKV) using correlation analysis. TKV was calculated by ultrasound, using the ellipsoid method.

**Results:** No difference in mean uNGAL levels was observed between groups (ADPKD: 26.36 ng/ml; Controls: 27.24 ng/ml; *P* = 0.96). Moreover, no correlation was found between uNGAL and proteinuria (*P* = 0.51), albuminuria (*P* = 0.69), TKV (*P* = 0.68), or mean arterial pressure (*P* = 0.90). By contrast, TKV was positively correlated with proteinuria (*P* = 0.04), albuminuria (*P* = 0.001), and mean arterial pressure (*P* = 0.03).

**Conclusion:** uNGAL did not confirm its superiority as a marker of disease progression in a pediatric ADPKD population. In the contrary, TKV appears to be an easy measurable variable and may be promising as a surrogate marker to follow ADPKD progression in children.

## Introduction

Autosomal dominant polycystic kidney disease (ADPKD) is the most prevalent inherited monogenic kidney disease and affects 1/500 individual worldwide. It represents up to 5–10% of total end-stage renal disease (1, 2). Its diagnosis is mainly based on renal ultrasonography anomalies due to the low cost, safety, and demonstrated reliability of this technique with 100% specificity and 67–95% sensitivity depending on the genetic mutation (3). Two genes are known to be involved in the disease. Mutations in *PKD1* gene concern around 85% of the ADPKD population and are associated with a more severe renal impairment and disease progression. Given the fewer cyst burden and later age at diagnosis among *PKD2*-related ADPKD, *PKD1*-associated ADPKD is over-represented in pediatric patients (4).

Until recently, ADPKD was considered mainly as an adult disease. However, clinical manifestations may already occur during early childhood. Developments in ultrasound technology have led to an increase in the prenatal and infant diagnosis of ADPKD (5) and, consequently, to the need for pediatric follow-up guidelines as clinical manifestations may already occur during early childhood.

Even in the early stages, 60% of ADPKD children present impaired urine concentration capacity several years before the decline in the glomerular filtration rate (GFR) (5). The estimated prevalence of proteinuria and albuminuria in ADPKD children is 20 and 30%, respectively (4, 6). Similar to the adult population, proteinuria has been shown to be associated with structural kidney changes in ADPKD pediatric patients (7). The prevalence of HTN in ADPKD children is 20% (8) and is also associated with a decreased GFR over time (1, 9).

Current biomarkers used to evaluate renal function in children at a stage of normal GFR highlight the difficulty for clinicians to determine whenever it is beneficial to introduce renal-protective agents. Albuminuria and proteinuria are routinely used as follow-up biomarkers in pediatric ADPKD patients, but they may not be present during the early stages of the disease. New biomarkers are required to detect the impact of the disease on kidney function. Glomerular hyperfiltration has been shown to be associated with a significantly faster decline in renal function and higher rate of kidney enlargement over time (10). In adults, Total Kidney Volume (TKV) has been recently studied as a surrogate marker of the disease progression and therapeutic efficacy (11–16). Neutrophil gelatinase–associated lipocalin (NGAL) is a small protein that consists of 178 amino acids produced by activated neutrophils and various epithelial cells with a rapid upregulated expression in response to any cellular stress, such as infection, inflammation, or ischemia (17, 18). Recent studies in adults have highlighted the interest of urinary NGAL (uNGAL) as a marker of acute kidney impairment (18, 19) as well as a possible biomarker of disease progression (17, 20, 21). Nishida et al. measured serum and uNGAL levels in patients with common pediatric renal diseases and demonstrated that uNGAL level was significantly increased compared to a control group, whether the GFR was normal or decreased. The increase was even more remarkable in tubular dysfunction disease (22).

Here we assessed the predictive value of uNGAL as an early tubular biomarker of renal function and impairment in ADPKD pediatric patients and compared values with a gender- and age-matched control group. We also investigated whether there was an association between uNGAL and well-recognized clinical markers of the disease, such as proteinuria, albuminuria, blood pressure, and TKV.

## Materials and Methods

### Population

Based on the data reported by Bolignano et al. (17) (estimated effect size: cohen's *d* = 3.73), we estimated that a sample size of 15 ADPKD patients and 15 controls would be sufficient to show uNGAL differences between groups.

The study was performed between December 2015 and July 2017 at Geneva University Hospitals (Geneva, Switzerland) among 15 ambulatory ADPKD patients aged <18 years old. The diagnosis of ADPKD was made using the typical ultrasound findings of renal cysts (i.e., presence of bilateral cysts) associated with a positive familial history. Two patients had been genetically tested before the study. Both of them had a *PKD1*-related mutation. None of the patients had acute kidney injury and any other congenital disease was an exclusion criterion. Data were collected for all patients during the regular annual follow-up consultation. A control group comprised of 15 ambulatory patients aged <18 years old recruited at a specialized consultation for pediatric voiding disorders was age and gender matched with the ADPKD patient group. The patients suffered diurnal and/or nocturnal enuresia due to behavioral issues. A normal kidney ultrasound was required as well as a normal urinary test (no presence of proteinuria or hematuria), and normal blood pressure. Any congenital disease was an exclusion criterion.

The protocol was approved by the local ethical committee of human research in Geneva (CER-15-230). The study was conducted in accordance with the principles of good clinical practice, the Declaration of Helsinki, and all local regulations. Written informed consent was obtained from parents and children >12 years old before participating in the study.

## Variables Analyses

### Urinary Biomarkers

NGAL measurement: 10 mL urine were collected on the day each participant came for an outpatient visit. The urine sample was therefore taken at a random time during day and was rapidly transferred to the laboratory where it was immediately centrifuged and stored at −80°C until assayed. NGAL was measured using an ELISA method on a commercial kit (KIT 036C; Bioporto®, Bioporto Diagnostics, Hellerup, Denmark) according to the manufacturer's instruction. All measurements were made in duplicate and in a blinded manner. NGAL levels were expressed as pg/mL and converted to ng/ml to make the comparison easier with the values found in the medical literature.

Proteinuria and albuminuria: in the ADPKD patient group, urine samples were also tested for albuminuria and proteinuria as standard care follow-up, calculated on the ratio of urinary albumin or total urinary proteins and urinary creatinine, respectively. Significant albuminuria was defined as >2.5 mg/mmol and significant proteinuria as >20 g/mol.

### TKV Assessment

All participants had a kidney ultrasound. In both groups, TKV (sum of right and left KV) was calculated upon the ultrasound image by the same pediatric radiologist using the ellipsoid method based on the length, width and depth (kidney volume = length × width × thickness × [π/6]) (23). The TKV unit is given as cm^3^. Two controls had their last normal ultrasound prior to the study and TKV was therefore not included in our analysis. The values in both groups were compared to standard kidney volume in children (24). The TKV was adjusted for height (in cm) in all subjects (Ht-TKV).

### Blood Pressure

Systolic and diastolic blood pressure were measured in both groups during the consultation and adjusted to age and height percentiles to determine the individual blood pressure percentile (25). A mean arterial pressure (systolic pressure + 2x diastolic pressure/3) was calculated in both groups.

### Statistical Analyses

Analyses were performed with the R software (26) including the BayesFactor package (27). We first checked normality using Shapiro-Wilk Test (28). We compared the concentration of uNGAL, which was not normally distributed using two-sample Wilcoxon tests. Categorical variable (i.e., Weight percentile, Height percentile, Systolic pressure percentile and Diastolic pressure percentile) were compared between groups using chi-squared test. Unpaired *t-*tests (Student *t*-test or Welch *t-*test for equal or unequal variance, respectively) were used to compare continuous variables between groups. When relevant, we confirmed null effects using JZS Bayes factor tests to estimate the ratio of the likelihood probability of the null and alternative hypothesis [i.e., Bayes factor (Bf) < 1 implies evidence for the null hypothesis]. We also checked for linear associations between uNGAL concentrations and clinical parameters of renal impairment, such as proteinuria, albuminuria, blood pressure and TKV, using Pearson's correlation. We used a significance level of alpha = 0.05.

## Results

Fifteen ADPKD patients (8 females; mean age: 10.2 years [standard deviation (SD): ±4.3; range: 4–17]) and 15 age- and gender-matched controls (8 females; mean age: 10.4 years [SD: ±3.4; range: 6–17]) participated in the study. No differences in age [*t*_(26)_ = 0.05; *P* = 0.96; Bf = 0.34], weight percentiles [χ^2^(6, *N* = 30) = 2.82, *P* = 0.83] or height percentiles [χ^2^(7, *N* = 30) = 9.96, *P* = 0.19] were observed between the two groups (Table 1). Patients' clinical characteristics are shown in Table 2.

**Table 1:** Patient and control group characteristics.

	**ADPKD**	**Controls**	***P*-Value**
*N*	15	15	
Age (y)	10.2 ± 4.3	10.4 ± 3.4	0.85
Gender (M/F)	7/8	7/8	1
Height (Percentile)			0.19
P3–P10	2	1	
P10–P25	3	4	
P25–P50	2	3	
P50–P75	5	3	
P75–P90	0	3	
P90–P97	3	0	
>P97	0	1	
Weight (Percentile)			0.83
P3–P10	1	2	
P10–P25	4	3	
P25–P50	2	2	
P50–P75	4	3	
P75–P90	3	2	
P90–P97	1	1	
>P97	0	2	

**Table 2:** Clinical characteristics of ADPKD patients.

**Subject**	**Age at diagnostic (year)**	**Familial history**	**Genetic mutation**	**Proteinuria >20 g/mol**	**Albuminuria >2.5 mg/mmol**	**Ungal (ng/ml)**	**TKV (cm^**3**^)**	**Ht-TKV (cm^**2**^)**	**Psyst percentile**	**Pdias percentile**
1	12	No	PKD1	Yes	Yes	25.038	426.9	2.55	P50–P90	P50–P90
2	5	Yes	Not tested	No	No	<5	217.8	1.19	<P50	<P50
3	<1	Yes	Not tested	Unknown	Unknown	<5	87.65	0.86	>P95 < P95 + 12 mmHg	P50–P90
4	9	Yes	Not tested	No	Yes	150.43	177.89	1.17	P50–P90	P50–P90
5	5	Yes	Not tested	No	No	104.38	177.51	1.27	P50–P90	<P50
6	7	Yes	Not tested	No	Yes	28.83	216.43	1.18	P50–P90	P50–P90
7	<1	Yes	Not tested	No	No	11.68	85.95	0.67	>P95 < P95 + 12 mmHg	<P50
8	<1	Yes	Not tested	No	No	34.96	194.46	1.25	P50–P90	P50–P90
9	*in utero*	Yes	Not tested	Yes	Yes	11.47	244.07	1.48	<P50	<P50
10	1	Yes	Not tested	Yes	No	<5	188.62	1.31	P50–P90	<P50
11	<1	Yes	Not tested	No	No	16.32	255.04	1.57	<P50	<P50
12	<1	Yes	PKD1	No	No	<5	75.9	0.78	P50–P90	P50–P90
13	6	Yes	Not tested	No	No	12.29	185.89	1.34	P50–P90	<P50
14	9	Yes	Not tested	Yes	Yes	<5	126.56	0.89	<P50	<P50
15	<1	Yes	Not tested	No	No	<5	110.23	1.05	P50–P90	P90–P95

*uNGAL, urinary NGAL; TKV, Total Kidney Volume; Ht-TKV, TKV corrected for height; Psyst, systolic pressure percentile adjusted for height and age; Pdias, diastolic pressure percentile adjusted for height and age*.

## Variables Analyses

### Urinary Biomarkers

**Figure 1:**
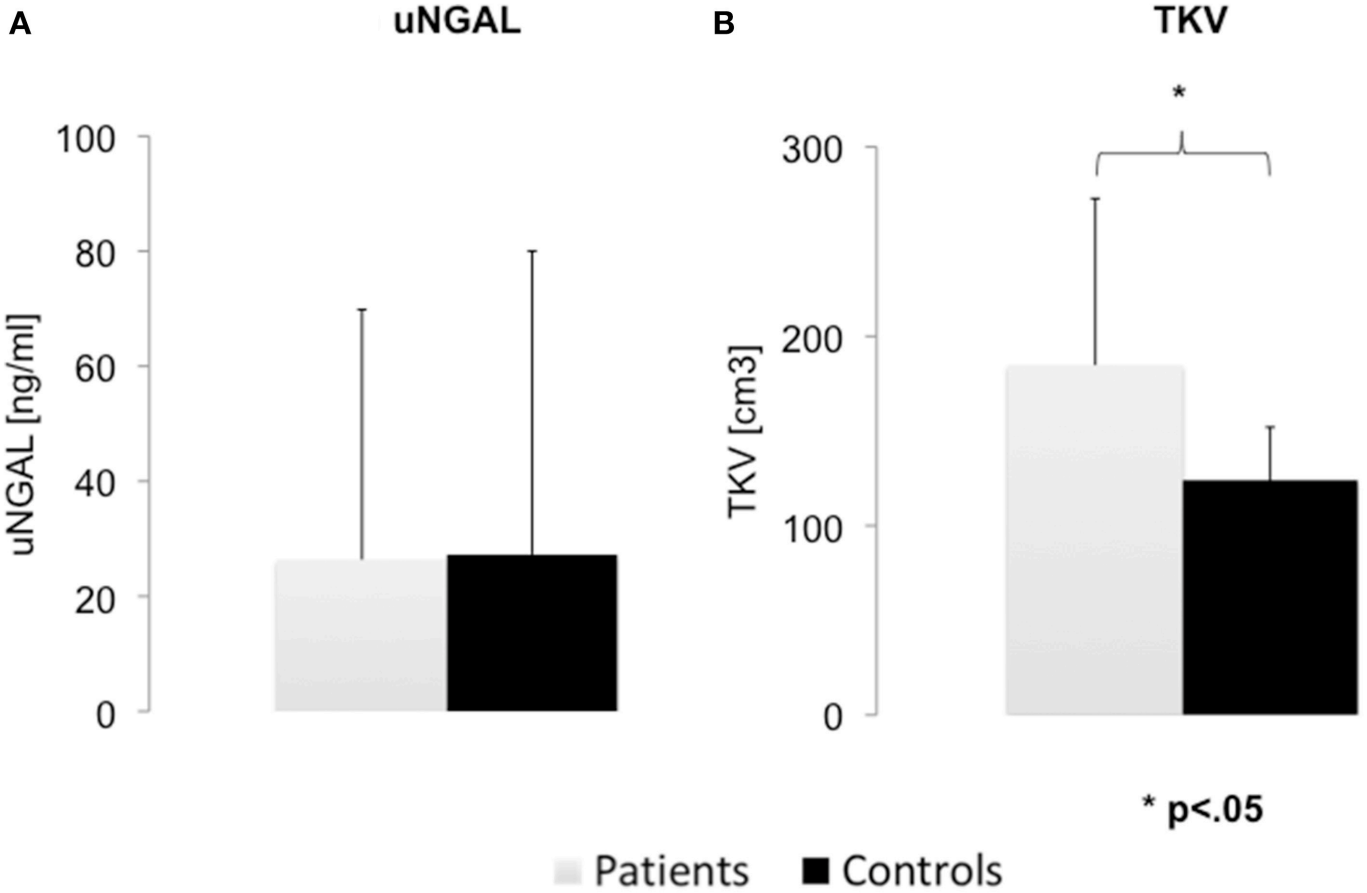
Difference in urinary NGAL and Total Kidney Volume between groups. **(A)** No differences in urinary NGAL (uNGAL) were observed between ADPKD patients and the gender and age matched control group. **(B)** Total kidney volume (TKV) was significantly higher in patients. Error bars show standard error of the mean.

uNGAL: uNGAL was not normally distributed (*p* < 0.001) unlike all other tested variables (all *p* > 0.2). Ten of 30 children (5 patients per group) had NGAL below the limits of detection defined as <5 ng/L (considered as zero for the calculation). No statistical difference in uNGAL was observed between patients (mean = 26.36 [ng/ml]; SD: ± 43.48; median = 11.68 [ng/ml]; IQR 0–27) and controls (mean = 27.24 [ng/ml]; SD: ± 52.78); (W = 109, *P* = 0.89; Bf = 0.34; Figure 1A).

Proteinuria and albuminuria: in the ADPKD group, 4 patients had a significant proteinuria and 5 had a significant albuminuria (>20 [g/mol] and >2.5 [mg/mmol], respectively; Table 2).

### TKV

TKV was calculated in the ADPKD group (mean: 184.73 [cm^3^]; SD; 88) and the control group (mean 123.83[cm^3^]; SD: 31) (Table 3). As predicted, TKV in the ADPKD group was significantly larger than in controls [*t*_(16)_ = 2.53; *P* = 0.02] (Table 3). When corrected for height, we detected a stronger difference between groups (ht-TKV ADPKD group: mean 1.24 [cm^2^]; SD; 0.44. Ht-TKV control group: mean 0.87 [cm^2^]; SD; 0.15) [*t*_(17)_ = 3.18; *P* = 0.005] (Figure 1B).

**Table 3:** Urinary NGAL, Total Kidney Volume and arterial pressure in percentile between groups.

	**ADPKD**	**Controls**	***P*-value**
*N*	15	15	
uNGAL [ng/ml]	26.36	27.34	0.96
TKV [cm^3^]	184.73	123.83	0.02
P*sys* (Percentile)			0.30
<P50	4	6	
P50–P90	9	9	
P90–P95	0	0	
>P95	2	0	
P*dias* (Percentile)			0.58
<P50	8	8	
P50–P90	6	7	
P90–P95	2	3	
>P95	0	0	

*uNGAL, urinary NGAL; TKV, Total Kidney Volume, Psys, systolic pressure; Pdias, diastolic pressure (arterial pressure percentiles adjusted for age and height percentile)*.

### Blood Pressure

Among the ADPKD group, 3 patients had systolic blood pressure, diastolic blood pressure or both >90th percentile when corrected for age and height percentiles at consultation (Table 2). None were taking any antihypertensive medication. All control group patients had blood pressure values <95th percentile. No differences between systolic blood pressure percentiles [χ^2^(2, *N* = 30) = 2.40, *P* = 0.30] or diastolic blood pressure percentiles [χ^2^(2, *N* = 30) = 1.08, *P* = 0.58] were observed between the two groups.

## Correlation Analyses

**Figure 2:**
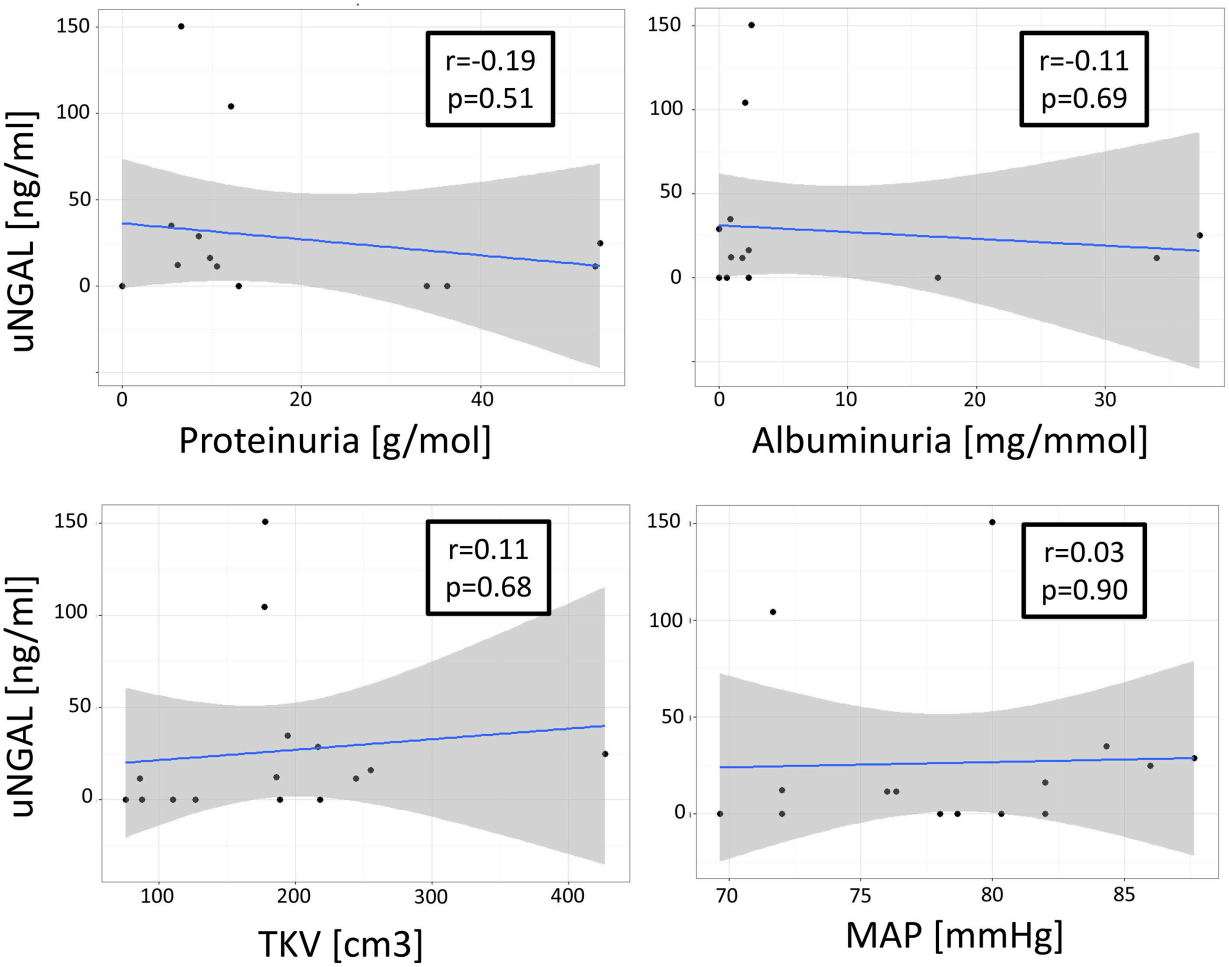
Association between uNGAL and parameters of renal impairment. No association shown between urinary NGAL (uNGAL) and Total Kidney Volume (TKV), mean arterial pressure (MAP), proteinuria [g/mol], or albuminuria [mg/mmol].

**Figure 3:**
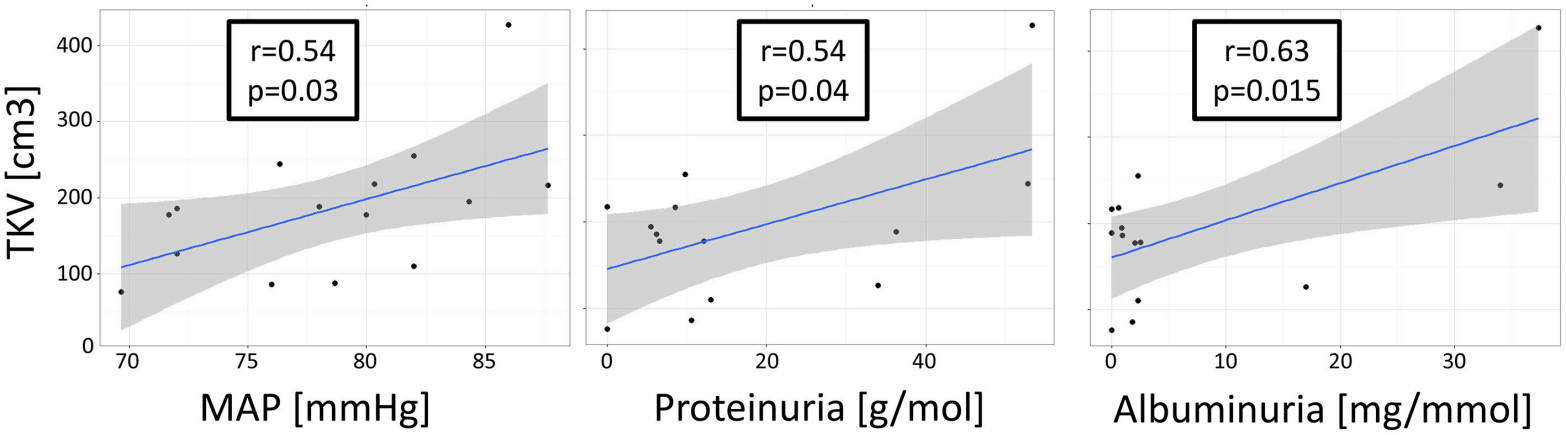
Association between TKV and parameters of renal impairment. Correlation analysis revealed a significant association between Total Kidney Volume (TKV), mean arterial pressure (MAP), proteinuria [g/mol], and albuminuria [mg/mmol].

Correlation analyses in the patient group revealed that the uNGAL concentration was not correlated with the TKV [*t*_(13)_ = 0.41; *r* = 0.11; *P* = 0.68], ht-TKV [*t*_(13)_ = 0.36; *r* = 0.10; *P* = 0.71], mean arterial pressure [*t*_(13)_ = 0.12; *r* = 0.03; *P* = 0.90], proteinuria [*t*_(12)_ = −0.67; *r* = −0.19; *P* = 0.51] or albuminuria [*t*_(12)_ = −0.41; *r* = −0.12; *P* = 0.69] (Figure 2). TKV and ht-TKV were associated with parameters of disease progression (Figure 3). They were significantly correlated with proteinuria [*t*_(12)_ = 2.25; *r* = 054; *P* = 0.04 and *t*_(12)_ = 2.47; *r* = 0.58; *P* = 0.02, respectively]. A similar relation was observed between albuminuria and TKV or ht-TKV [*t*_(12)_ = 2.82; *r* = 0.63; *P* = 0.001 and *t*_(12)_ = 2.91; *r* = 0.64; *P* = 0.001, respectively] as well as mean arterial pressure [*t*_(13)_ = 2.31; *r* = 0.54; *P* = 0.03 and *t*_(13)_ = 1.91; *r* = 0.47; *P* = 0.07, respectively] (Figure 3).

## Discussion

Urinary Neutrophil Gelatinase-Associated Lipocalin (uNGAL) is frequently proposed as an early marker for renal progression in ADPKD adult patients. Similar to a previous study conducted in the adult ADPKD population by Vareesangthip et al. (29), we found no significant difference in uNGAL values between the two groups. This finding is in contrast to our hypothesis and other observations in adults (17, 20), reporting higher uNGAL values when compared to controls. An explanation that may account for this discrepancy is the possibility that uNGAL concentration is significantly higher only when GFR has already declined. Bolignano et al. found a significant difference between uNGAL in ADPKD patients compared to controls, but the mean creatinine clearance in the ADPKD group was 59 ± 38 ml/min, which is already a consistent decrease in renal function (17). It implies that uNGAL may be useful as a biomarker during advanced stages of ADPKD. Moreover, the importance of the cystic disease on uNGAL excretion may not be as important as reported. Parikh et al. (30) demonstrated that even though uNGAL was present and highly expressed in the epithelial cells of kidney cysts and its excretion increased in ADPKD patients, it did not correlate with a worsening of kidney function or an increase in TKV. However, as hypothesized by the authors, these results may be due to the lack of communication between the cystic space and the urinary collection system (30). On the other hand, several studies showed a positive correlation between uNGAL values and a worsening of renal function or creatininemia (17, 21, 29).

Kawano et al. (20) have explored potential urinary biomarkers and showed that NGAL could be a possible common biomarker for human and murine ADPKD as it was significantly higher than in the control group. Among 26 ADPKD adult patients, Bolignano et al. (17) demonstrated a significant increase in serum and uNGAL levels compared to a control group. Moreover, in the ADPKD group, patients with higher cystic growth presented higher serum and uNGAL levels. Plasma and uNGAL levels were shown to correlate with both a residual GFR and creatininemia, suggesting that the levels of this protein may in some way be influenced by the degree of the underlying altered renal function. A study by Meijer et al. among 102 ADPKD adult patients demonstrated that uNGAL was not only increased in the patient group compared to the control group, but that it was also associated with GFR, renal blood flow and TKV. Interestingly, these associations were independent of albuminuria (21). Nishida et al. reported a significant increase in uNGAL levels than in serum NGAL in patients with several common pediatric renal diseases compared to a control group of healthy children (22).

In our ADPKD group, uNGAL concentrations were compared to other clinical markers used to assess disease progression in children, such as proteinuria, albuminuria, mean arterial pressure, and TKV. No correlation was found between albuminuria, proteinuria, and uNGAL. However, significant proteinuria and albuminuria concerned only a few of our patients. Previous studies in children and adults demonstrated that proteinuria and albuminuria were associated with a more rapid progression toward more severe renal disease (5). HTN is a common complication of the disease that may already occur during childhood. In our study, systolic, diastolic, and mean arterial pressure, were compared between groups. No differences between systolic blood pressure percentiles or diastolic blood pressure percentiles were observed between the two groups.

Interestingly, we found a significant correlation between proteinuria and TKV/Ht-TKV and between albuminuria and TKV/Ht-TKV. These findings are consistent with a previous study in 100 young patients (mean age: 31 years) with ADPKD and preserved renal function where the degree of albuminuria correlated with TKV and kidney volume growth rate, measured by magnetic resonance imaging sequences (31). Similarly, in their study among 103 ADPKD children, Sharp et al. showed that those with severe renal cystic disease (>10 cysts; *n* = 54) had greater protein excretion than patients with moderate disease (< or = 10 cysts; *n* = 49) (6). When comparing mean arterial pressure and TKV, a significant correlation was found. This is consistent with Seeman et al. who demonstrated a correlation between HTN and the number and volume of cysts in a study of 62 children suffering from ADPKD (32).

TKV has been recently demonstrated as a marker of disease progression and therapeutic efficacy (11–16) in the adult ADPKD population. Among 85 patients with ADPKD aged 4–21 years and followed for 5 years, Cadnapaphornchai et al. were able to demonstrate a strong association between TKV increase and hypertension (9). Various imaging modalities such as magnetic resonance imaging, computed tomography, or ultrasonography can quantify TKV. One simple, practical and non-invasive standardized method to estimate TKV is based on ultrasound with the use of the ellipsoid formula (23, 33). Sonographic measurement of kidney volume in adults with ADPKD was compared to magnetic resonance imaging measurements. Although it was demonstrated to be inappropriate for the evaluation of short-term disease progression, it was sufficient to estimate kidney volume, which reflects disease severity (34). Among 30 ADPKD children, Breysem et al. demonstrated a significant correlation between magnetic resonance imaging and ultrasound TKV measurements. However, there was a strongest correlation using a 3D vs. 2D ultrasound measurement (33). Data from the Consortium for Radiologic Imaging Studies of Polycystic Kidney Disease (CRISP) study have shown that increases in Ht-TKV are associated with more frequent and severe complications of ADPKD, such as hypertension, gross hematuria, and proteinuria, and also loss of kidney function (13–16). Moreover, Yu et al. recently reported that not only basal kidney volume, but also the rate of kidney growth, are strongly associated with the development of advanced stages of kidney disease in ADPKD patients (35). These findings are consistent with the possible use of TKV as a predictive and potentially monitoring biomarker in ADPKD.

In our study, we could not demonstrate a correlation between TKV/Ht-TKV and uNGAL concentration. This result is consistent with a previous study conducted by Petzold et al. on 139 ADPKD patients considered at an early stage of the disease (mean estimated GFR: 93 ± 19 ml/min/1.73m^2^). Different urinary biomarkers were dosed and tested for an association with the estimated GFR and Ht-TKV. No association was found between uNGAL and the estimated GFR or Ht-TKV (36). The fact that no association was found at an early stage of the disease in this adult population may explain our results. Moreover, this result may support the hypothesis of a lack of communication between the cystic space and the urinary collection system as previously mentioned. However, our results are in contrast with Meijer et al. who demonstrated a positive correlation between uNGAL and TKV (21) in adult ADPKD patients. Bolignano et al. found that ADPKD patients with higher cyst growth had higher serum and uNGAL (17).

To our knowledge, this is the first study investigating the clinical relevance of uNGAL in an ADPKD pediatric population. Our study has some limitations though. Firstly, is the relative small sample size that could theoretically have hidden a difference in uNGAL between groups. However, our power analysis suggests that our study was not underpowered. Moreover, to maximize the sensitivity of our test, the uNGAL values in the ADPKD children were compared with a carefully selected age and gender matched control group. Finally, bayesian analysis confirmed the absence of effect and provided compelling evidence for the null hypothesis compared to the alternative hypothesis (Bf = 0.39). Bring together, these observations strongly support the idea that more subjects would not have led to a significant result. Another limitation is the variability in uNGAL values observed between participants. We argue that this cannot explain the absence of a significant difference between groups for the following reasons. First, the absence of a significant difference persisted following outlier removal, using a well-described method for data exclusion (1.5-fold IQR). Second, the uNGAL values in our population were within the normal range values proposed by Bennett et al. (37). Thus, the variability of uNGAL among individuals seems to compromise its use as biomarker of renal impairment in the early stages of the disease. Some other limitations have to be addressed. Firstly, uNGAL was measured at a single time point and not longitudinally. We cannot therefore comment on changes in uNGAL concentration over time, limiting the evaluation of its potential use as marker of the disease progression. In addition, urine samples were collected during the day and not at first morning void, not considering the variability of proteinuria during the day.

GFR is usually normal in children with ADPKD (38, 39). In our study GFR was not measured. Consequently, correlation between GFR and uNGAL or any other marker of renal impairment was not studied. Even if these correlation analyses could have led to a better comprehension of the markers modified by the disease progression, considering the absence of difference in uNGAL between groups, it would not have modified our conclusion.

Finally, our ADPKD population was not genetically tested to identify *PKD1* or *PKD2* mutation. We can assume that *PKD1* was predominant in our patients as patients with *PKD2* mutation express cysts later and *PKD1* is over-represented in pediatric ADPKD population (4).

## Conclusions

Our results demonstrate the absence of interest of uNGAL as an early biomarker of renal function in the pediatric population. Interestingly, we showed a significant correlation between TKV and markers of disease progression, such as proteinuria, albuminuria, and mean arterial pressure. TKV is easy, measurable and low cost and may be a surrogate marker to follow ADPKD progression disease in children. More studies on early markers of renal impairment and disease progression in children are needed.

## Data Availability

The datasets generated for this study are available on request to the corresponding author.

## Author Contributions

MT and PP designed the study. MT, AW-B, and AG developed the methodology and collected the data. MT, MS, LM, and PP performed the analysis, and interpreted the results. MT and PP wrote the initial draft of the paper and all authors provided critical review, edits, and approval for the final manuscript.

### Conflict of Interest Statement

The authors declare that the research was conducted in the absence of any commercial or financial relationships that could be construed as a potential conflict of interest.
